# Sonometric assessment of cough predicts extubation failure: SonoWean—a proof-of-concept study

**DOI:** 10.1186/s13054-023-04653-w

**Published:** 2023-09-25

**Authors:** Vincent Bonny, Jeremie Joffre, Paul Gabarre, Tomas Urbina, Louai Missri, Mathilde Ladoire, Maxime Gasperment, Jean-Luc Baudel, Bertrand Guidet, Guillaume Dumas, Eric Maury, Laurent Brochard, Hafid Ait-Oufella

**Affiliations:** 1 Service de Médecine Intensive Réanimation, Assistance Publique - Hôpitaux de Paris (AP-HP), Hôpital Saint-Antoine, 184 Rue du Faubourg Saint-Antoine, 75571 Paris Cedex 12, France; 2 Sorbonne Université, Paris, France; 3 Service de Médecine Intensive-Réanimation, CHU Grenoble-Alpes, INSERM U1042-HP2, Université Grenoble-Alpes, Grenoble, France; 4 Unity Health Toronto, Keenan Research Centre, Interdepartmental Division of Critical Care Medicine, Li Ka Shing Knowledge Institute, St. Michael’s Hospital, University of Toronto, Toronto, ON 508783 Canada; 5 Inserm U970, Centre de Recherche Cardiovasculaire de Paris (PARCC), Paris, France

**Keywords:** Weaning, Mechanical ventilation, Cough, Extubation failure, Sonometric

## Abstract

**Background:**

Extubation failure is associated with increased mortality. Cough ineffectiveness may be associated with extubation failure, but its quantification for patients undergoing weaning from invasive mechanical ventilation (IMV) remains challenging.

**Methods:**

Patients under IMV for more than 24 h completing a successful spontaneous T-tube breathing trial (SBT) were included. At the end of the SBT, we performed quantitative sonometric assessment of three successive coughing efforts using a sonometer. The mean of the 3-cough volume in decibels was named Sonoscore.

**Results:**

During a 1-year period, 106 patients were included. Median age was 65 [51–75] years, mainly men (60%). Main reasons for IMV were acute respiratory failure (43%), coma (25%) and shock (17%). Median duration of IMV at enrollment was 4 [3–7] days. Extubation failure occurred in 15 (14%) patients. Baseline characteristics were similar between success and failure extubation groups, except percentage of simple weaning which was lower and MV duration which was longer in extubation failure patients. Sonoscore was significantly lower in patients who failed extubation (58 [52–64] vs. 75 [70–78] dB, *P* < 0.001). After adjustment on MV duration and comorbidities, Sonoscore remained associated with extubation failure. Sonoscore was predictive of extubation failure with an area under the ROC curve of 0.91 (IC_95%_ [0.83–0.99],* P* < 0.001). A threshold of Sonoscore < 67.1 dB predicted extubation failure with a sensitivity of 0.93 IC_95%_ [0.70–0.99] and a specificity of 0.82 IC_95%_ [0.73–0.90].

**Conclusion:**

Sonometric assessment of cough strength might be helpful to identify patients at risk of extubation failure in patients undergoing IMV.

**Supplementary Information:**

The online version contains supplementary material available at 10.1186/s13054-023-04653-w.

## Introduction

Invasive mechanical ventilation (IMV) is a common support organ therapy in intensive care unit (ICU). Given that prolonged IMV is associated with increased mortality, its duration has to be as limited as possible to reduce exposure to IMV-related complications [1, 2]. Nevertheless, extubation failure (EF) is also associated with a significant increase in mortality. It is therefore of paramount importance to develop easy-to-use tools at the bedside to evaluate the risk of EF [3].

Several trials suggested that ineffective cough is associated with EF in patients undergoing IMV owing to impaired airway clearance leading to secretion accumulation, atelectasis and secondary respiratory tract infection [4, 5]. An International Consensus Conference published in 2007 [6] highlighted that appropriate cough is required for extubation but the optimal way to assess cough efficiency is still debated [7, 8].

In this study, we hypothesized that quantitative sonometric assessment of the cough in patients who successfully passed a spontaneous breathing trial (SBT) might help to identify those at high risk of EF.

## Methods

### Population and weaning protocol

We conducted a prospective observational study in an 18-bed medical ICU in France (Saint-Antoine Hospital, Assistance Publique–Hôpitaux de Paris) from October 2021 to October 2022. Every adult patient requiring IMV for more than 24 h was eligible. According to local practice, standard oxygen therapy was the only oxygen delivery strategy applied after extubation. Exclusion criteria were limited to patients unable to cough 3 times to order, patients exhibiting an upper airway obstruction or requiring a reintubation for surgery or endoscopy. Only the extubation following the first successful SBT was solely analyzed. Ready-to-wean criteria and SBT failure criteria relied on guidelines from the International Consensus Conference on Weaning [6]. Prophylactic non-invasive ventilation (NIV) was standardized and used if patients were at high risk of EF as proposed by Rochwerg et al. [9].

The primary outcome was extubation failure, defined as reintubation within 48 h after extubation or as initiation of unplanned NIV or curative NIV in patients already receiving prophylactic NIV within the 48 h after extubation. Indication for reintubation, unplanned or curative NIV was post-extubation acute respiratory failure, defined by the presence of one or more of the following criteria, persistent over 5 min: SpO2 < 90% with an oxygen support ≥ 5 L/min, a respiratory rate ≥ 35/min, a pH < 7.35 with a pCO2 > 45 mmHg and/or use of accessory respiratory muscles. High flow nasal oxygen (HFNO) use was let at the clinician’s discretion.

### Cough assessment

At the end of successful SBT, once considered ready for extubation by the attending physician, measurement was performed after endotracheal aspiration just before extubation. Cough strength, estimated by its sound level (decibels), was assessed by Pulsar Model 14® Sound-Level Meter (Additional file 1: Fig. S1) placed 2 cm away from the distal part of the endotracheal tube. Patients were asked to cough three times in a row in a maximum effort, allowing estimating the Sonoscore (*i.e., *mean in decibel produced by the three consecutive coughing efforts). Simultaneously, cough effectiveness was subjectively evaluated by the caregivers including nurse, resident and senior physician in charge of the patient using a three-levels scale (0, ineffective cough; 1, moderate cough; 2, effective cough). All caregivers were blinded to Sonoscore throughout the study.

**Fig. 1:**
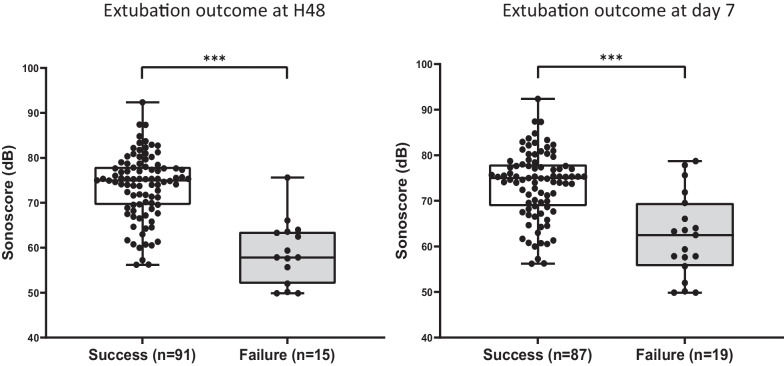
Sonoscore according to extubation outcome at H48 (left) and Day 7 (right). Box plots showing patient’s Sonoscore according to extubation outcome. Data are presented as median [interquartile range], ***P < 0.001, Mann–Whitney test. *dB* Decibels

### Statistical analysis

Continuous variables were expressed as median [IQR], and categorical variables were expressed as absolute and relative frequencies. To assess associations between patient characteristics and EF, we first performed univariate analyses based on the two-tailed Mann–Whitney test or chi-squared test as appropriate. To identify independent predictors of EF, variables with *P* values less than 0.05 by univariate analysis and known factors associated with EF were included in a multivariable logistic regression model. The Kolmogorov–Smirnov test was used to evaluate the normality distribution of the continuous variables. The model's goodness of fit was assessed using the Hosmer–Lemeshow test and the discrimination by the area under the receiver operating characteristic curve (ROC-AUC). We used Youden’s method to determine the best cut-off predictive value of Sonoscore for EF. All tests were two-sided, and *P* values less than 0.05 were considered statistically significant.

## Results

### Study population

From October 2021 to October 2022, 114 patients met the inclusion criteria. Of these, 8 patients were excluded from the analysis (Additional file 1: Fig. S2) and 106 patients were finally included, mainly male (60%), with a median age of 65 [51–75] years. The main characteristics at admission are depicted in Table 1.

**Fig. 2:**
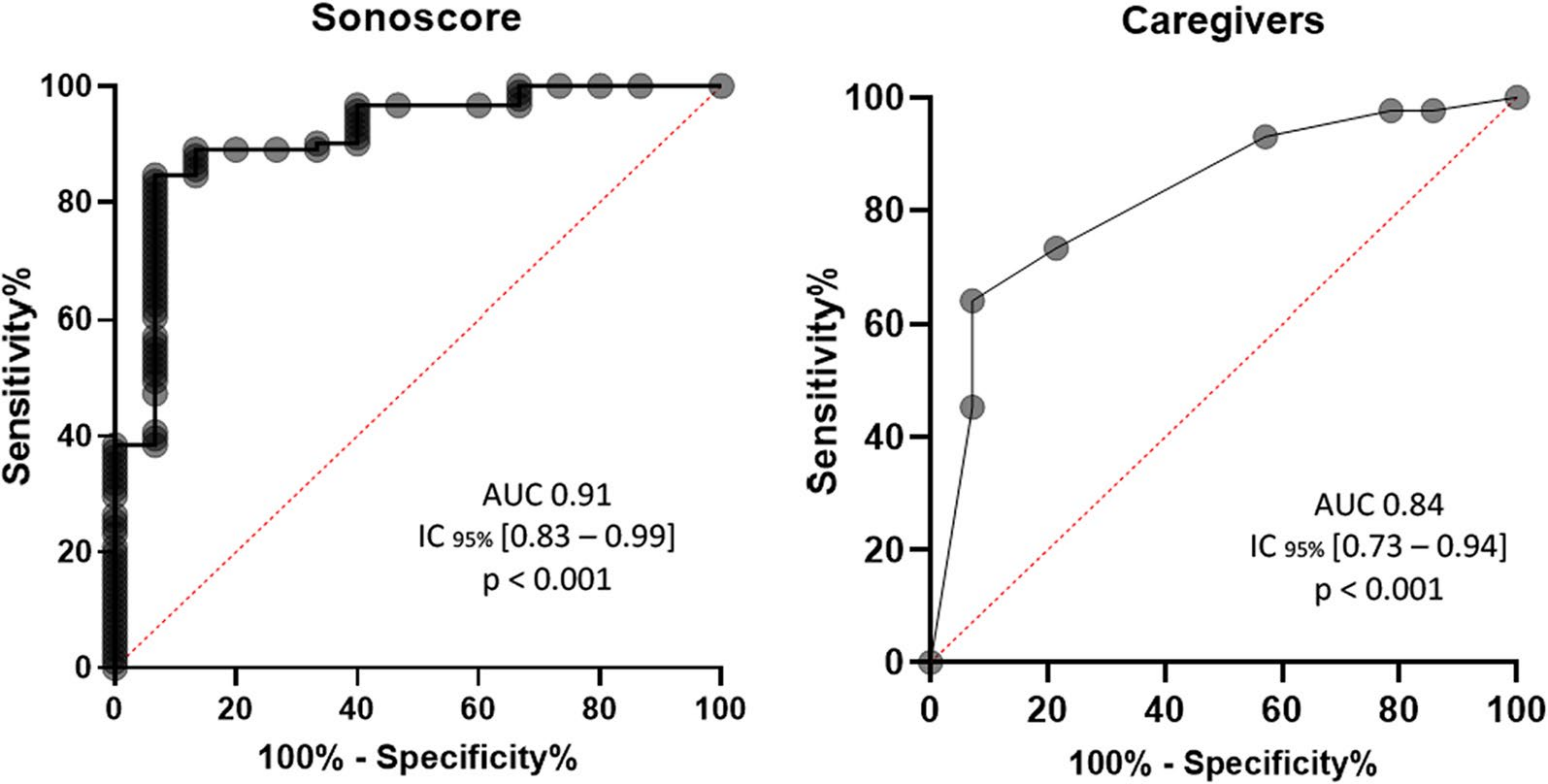
Performance of the Sonoscore (left) and the caregivers’ subjective assessment (right) of cough to predict extubation failure. The Sonoscore with an area under the curve (AUC) significantly higher (*P* = 0.014, Delong’s test) than the caregiver’s subjective assessment of cough

**Table 1:** Patients’ characteristics at inclusion

	Overall population (*n* = 106)	Extubation success (*n* = 91)	Extubation failure (*n* = 15)	*P*
Characteristics at admission				
Age, years	65 [51–75]	64 [48–74]	68 [54–77]	0.671
Male sex, *n* (%)	64 (60)	55 (60)	9 (60)	> 0.999
Body-mass index, kg/m2	28 [22–29]	26 [22–29]	24 [21–28]	0.223
SAPS II, points	48 [37–62]	48 [36–60]	55 [41–70]	0.216
SOFA score, points	6 [4–9]	6 [4–9]	7 [3–10]	0.194
Medical history, *n* (%)				
Underlying chronic cardiac disease	28 (26)	25 (26)	3 (20)	0.754
History of cancer	26 (25)	22 (24)	4 (27)	0.512
Underlying chronic lung disease	20 (19)	18 (20)	2 (13)	0.731
Neuromuscular disease	4 (4)	2 (23)	2 (13)	0.095
Main reason for intubation, *n* (%)				
Acute respiratory failure	46 (43)	38 (42)	8 (53)	0.416
Coma	27 (25)	25 (27)	2 (13)	0.345
Shock	18 (17)	14 (15)	4 (27)	0.280
Cardiac arrest	6 (6)	5 (6)	1 (7)	> 0.999
Other	9 (9)	9 (10)	0	0.353
Ventilatory characteristic at extubation				
Duration of mechanical ventilation, days	4 [3–7]	4 [3–7]	8 [3–12]	**0.016**
Weaning difficulty				
Simple, *n* (%)	69 (65)	63 (69)	6 (40)	**0.040**
Difficult, *n* (%)	26 (25)	23 (25)	3 (20)	> 0.999
Prolonged, *n* (%)	11 (10)	5 (6)	6 (40)	**0.001**
Pressure-support level, cmH2O	10 [7–12]	10 [7–12]	9 [7–12]	0.915
PEEP, cmH2O	5 [5–6]	5 [5–6]	5 [5–6]	0.410
Tidal volume, ml	427 [379–503]	427 [380–514]	415 [333–461]	0.250
Respiratory rate, breaths/min	24 [19–30]	24 [19–29]	27 [20–40]	0.293
FiO2, mmHg	30 [25–40]	30 [25–40]	30 [25–40]	0.983
pH, units	7.46 [7.42–7.50]	7.46 [7.42–7.50]	7.46 [7.42–7.53]	0.619
PaCO2, mmHg	36 [31–41]	37 [32–42]	35 [30–40]	0.292
Abundant secretions, *n* (%)	18 (17)	15 (16)	3 (20)	0.717

Bold symbol was used in case of P value < 0.05

Data are presented as median [interquartile range] or number (percentage)

*SAPS* Simplified Acute Physiology Score; *SOFA* Sequential Organ Failure Assessment; *PEEP* positive end-expiratory pressure; *FiO2* fraction of inspired oxygen; *PaCO2* partial pressure of arterial carbon dioxide

### Extubation outcome

Extubation failure occurred in 15 (14%) patients, 8 were reintubated and 7 required NIV (4 patients with unplanned NIV and 3 patients with curative from prophylactic NIV, with a median duration of 96 [58–114] hours). Baseline clinical and biological characteristics were similar between extubation success and failure groups, except for 2 parameters: the ratio of simple weaning which were lower and the duration of IMV longer in failure patients (Table 1).

### Sonometric assessment of cough

Sonoscore was significantly lower in patients who failed extubation at H48 (with 58 [52–64] vs.75 [70–78] dB, median difference - 17 [- 19–- 11] dB, *P* < 0.001). Similar results were observed when extubation outcome was evaluated at day 7 (Fig. 1). Dynamic of cough was of interest because cough-related sound level significantly increased between the first and the third coughing effort (median level 73 [66–78] vs*.*76 [71–80] dB, median difference + 3 [1–5] dB, *P* < 0.001) in the success group whereas sound level did not change and remained low in patients who failed extubation (median level 59 [52–63] *vs.*60 [54–64] dB, median difference + 1 [0–4] dB, *P* = 0.187) (Additional file 1: Fig. S3). The Sonoscore did not change according to the duration of IMV.

Sonoscore was an accurate tool to predict EF with an AUC at 0.91 IC_95%_ [0.83–0.99] *P* < 0.001), significantly higher (*P* = 0.014, Delong’s test) than the caregiver’s subjective assessment of cough (AUC 0.84 IC_95%_ [0.73–0.94] *P* < 0.001) (Fig. 2). A threshold of Sonoscore at 67.1dB predicted EF with a sensitivity of 0.93 IC_95%_ [0.70–0.99] and a specificity of 0.82 IC_95%_ [0.73–0.90]. In a multivariate logistic regression model including mechanical ventilation duration in days and presence of cardiac and/or lung disease, Sonoscore remained independently associated with extubation failure (OR = 51.9 [9.05–988], *P* < 0.001) (Additional file 1: Fig. S4).

## Discussion

In a monocentric prospective cohort including ICU patients under IMV, we found that quantitative assessment of cough using sonometry was a promising tool to identify patients at high risk of extubation failure.

Weaning from mechanical ventilation is a crucial step in the management of ICU patients, representing up to 50% of total duration of IMV [10, 11]. Decision to extubate is of paramount importance because post-extubation acute respiratory failure requiring mechanical ventilation is associated with significantly higher mortality [12]. Previous works have shown that EF is related to a respiratory cause in more than 50% of cases, ineffective cough and weakness of respiratory muscles being reported as the main drivers for reintubation [13]. Effective cough as important criteria for extubation has been proposed by experts [6], but its quantitative assessment remains difficult. Cough has been evaluated using a peak flow meter (*i.e.,*pneumotachograph or electronic flowmeter) but its predictive value for EF is not optimal (sensitivity of 79% and specificity of 71%) [14, 15]. In our work, we used a sonometer device allowing accurate quantification of the sound generated during cough effort assuming that the sonometric assessment of the cough is a surrogate of its strength. Here, we showed that a standardized sonometric evaluation of the cough at the bedside and more specifically the Sonoscore may provide additional insight from caregivers’ assessment to identify more accurately patients at risk of EF. Weaning process should aim to discriminate whether patients are ready to be extubated or not. In order to avoid the excess mortality associated with EF, we chose a Sonoscore threshold at 67.1 dB to predict EF with the best sensitivity/specificity combination as well as the highest sensitivity.

The duration of IMV positively correlates with respiratory muscle weakness [16, 17]. However, in our study, we did not find any association between the Sonoscore and the duration of MV. This suggests the role of a pre-existing inability to ensure a strong cough in EF patients, which would not be entirely explained by ICU-acquired weakness alone. Most studies focus on inspiratory muscle dysfunction during weaning [18–20], while abdominal wall muscles play an important role in active expiration and cough effectiveness. In contrast, the impact of IMV on expiratory muscles function has been less investigated and interventions targeting these muscles are scarce.

Our study has several limitations. First, the sample size is limited and results have to be confirmed in a larger cohort. Second, sonometric assessment of cough has been performed on a T-piece SBT, but its predictive value in patients with pressure-support ventilation SBT needs to be evaluated. Third, although no difference was observed regarding the abundance of secretions between patients with extubation success or failure, whether sonometric assessment of cough is affected in hypersecretive patients remains unclear. Fourth, cough assessment requires the patient’s cooperation. Fifth, despite its good prognostic value, Sonoscore does not provide pathophysiological information on the mechanism leading to extubation failure. Finally, in our study, we did not measure cough peak flow. Comparing the predictive value of Cough Peak Flow *versus* sonometric assessment should be evaluated in a future work.

## Conclusion

In a monocentric prospective cohort including non-selected ICU patients under IMV who succeeded SBT, we found that objective cough assessment using sonometry may help to identify patients at high risk of extubation failure. Those results must be validated in a larger cohort.

### Supplementary Information

**Additional file 1. Supplemental Fig 1**: Description of the Pulsar Model 14® Sound Level Meter and method for measurement. The Model 14 is a general purpose digital sound level meter which meets the full requirements of IEC 61672 to Class 2. Before each inclusion the Sound Level Meter was calibrated acoustically using an external reference, i.e the Sound Level Calibrator Model 106, which is placed over the microphone. The calibrator generates a stabilized Sound Pressure Level of 94dB (+- 0.3dB) at a frequency of 1 kHz. Using a Low range (Low = 35dB to 100dB), maximum sound level was measured pressing the MAX HOLD button for at least ½ second and was ultimately noticed. A level of sound in decibels (L) is defined as ten times the base-10 logarithm of the ratio between two power-related quantities I (i.e cough-volume related sound) and Io (i.e the human hearing threshold) as follows: L = 10 * Log 10 (I/ Io). Thus, an apparent mild increase from 73 to 76 dB in sound level results in multiplying acoustic energy by a factor two.

## Data Availability

The datasets used and/or analyzed as well GraphPad sheets used in the current study are available from the corresponding author upon reasonable request.

## Abbreviations

IMV: Invasive mechanical ventilation
ICU: Intensive care unit
EF: Extubation failure
SBT: Spontaneous breathing trial
NIV: Non-invasive ventilation
dB: Decibel
IQR: Inter-quartile range
ROC: Receiver operating characteristic
AUC: Area under the curve
SAPS: Simplified acute physiology score
SOFA: Sequential organ failure assessment
PEEP: Positive end-expiratory pressure
FiO2: Fraction of inspired oxygen
PaCO2: Partial pressure of arterial carbon dioxide
