# Supplementary material for: Sonometric assessment of cough predicts extubation failure: SonoWean—a proof-of-concept study
Source: Crit Care. 2023 Sep 25;27:368. doi: 10.1186/s13054-023-04653-w (PMC10521471; doi:10.1186/s13054-023-04653-w)
Supplement: Supplementary file 1 — Additional file 1. Supplemental Fig 1: Description of the Pulsar Model 14® Sound Level Meter and method for measurement. The Model 14 is a general purpose digital sound level meter which meets the full requirements of IEC 61672 to Class 2. Before each inclusion the Sound Level Meter was calibrated acoustically using an external reference, i.e the Sound Level Calibrator Model 106, which is placed over the microphone. The calibrator generates a stabilized Sound Pressure Level of 94dB (+- 0.3dB) at a frequency of 1 kHz. Using a Low range (Low = 35dB to 100dB), maximum sound level was measured pressing the MAX HOLD button for at least ½ second and was ultimately noticed. A level of sound in decibels (L) is defined as ten times the base-10 logarithm of the ratio between two power-related quantities I (i.e cough-volume related sound) and Io (i.e the human hearing threshold) as follows: L = 10 * Log 10 (I/ Io). Thus, an apparent mild increase from 73 to 76 dB in sound level results in multiplying acoustic energy by a factor two. [file 13054_2023_4653_MOESM1_ESM.docx]

**Supplemental Figure 1**: Pulsar Model 14® Sound Level Meter


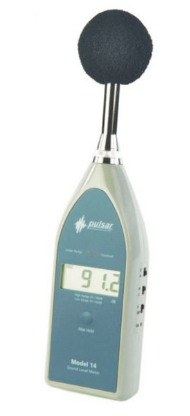


**Supplemental Figure 1**: Description of the Pulsar Model 14® Sound Level Meter and method for measurement. The Model 14 is a general purpose digital sound level meter which meets the full requirements of IEC 61672 to Class 2. Before each inclusion the Sound Level Meter was calibrated acoustically using an external reference, *i.e* the Sound Level Calibrator Model 106, which is placed over the microphone. The calibrator generates a stabilized Sound Pressure Level of 94dB (+- 0.3dB) at a frequency of 1 kHz. Using a Low range (Low = 35dB to 100dB), maximum sound level was measured pressing the MAX HOLD button for at least ½ second and was ultimately noticed.

A level of sound in decibels (L) is defined as ten times the base-10 logarithm of the ratio between two power-related quantities I (i.e cough-volume related sound) and Io (i.e the human hearing threshold) as follows: L = 10 * Log 10 (I/ Io). Thus, an apparent mild increase from 73 to 76 dB in sound level results in multiplying acoustic energy by a factor two

**Supplemental Figure 2**: Flow Chart.

114 patients undergoing a spontaneous breathing trial

4 patients excluded from analysis:

- 2 upper airway obstructions
- 2 intubated for surgery

110 patients eligible for analysis

4 patients unable to cough 3 times

106 patients analyzed

91 extubation success

15 extubation failure

- 8 requiring intubation
- 7 NIV +/- HFNO

**Supplemental Figure 2 legend:** Abbreviation: NIV, non-invasive ventilation; HFNO, High-Flow Nasal Oxygen

**Supplemental Figure 3:** Sound level of the first and last cough effort according to extubation outcome.

**Supplemental Figure 3 legend:** Data are presented as median [interquartile range], *** *P* < 0.001, paired non-parametric test. Abbreviation: dB, Decibels.

**Supplemental Figure 4:** Multivariate regression analysis of factors associated with extubation failure.


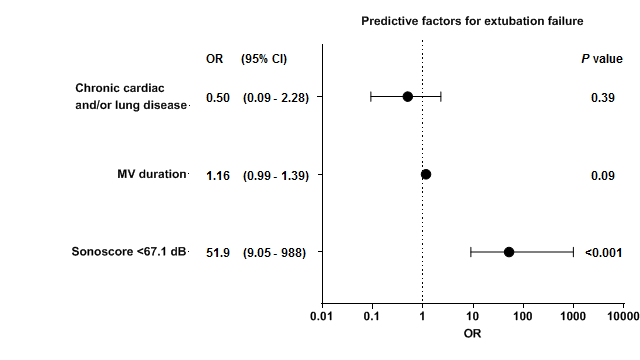


**Supplemental Figure 4 legend:** The dots represent the odds ratio; dot size is proportional to the odds ratio. The line through each dot corresponds to the 95% confidence interval. Calibration (AUC-ROC): 0.93 (0.88-0.98); P<0.0001. Goodness of fit (Hosmer-Lemeshow statistic): P value = 0.86. Abbreviation: MV duration, Mechanical ventilation duration (days); dB, decibel; OR, Odds ratio.
